# Artificial Anus

**Published:** 1828-09

**Authors:** 


### ARTIFICIAL ANUS.

### *Case of Artificial Anus, treated at the* Glasgow Infirmary, *by* Dr. Anderson.

Mary Stewart, admitted 10th November. In the left groin, there was a very large sore, extending from the spine of the ilium to the symphysis pubis. It was about three inches broad, passing above Poupart's ligament, of a ragged, irregular, and sloughy appearance, and having a sinus running by the side of the labium down towards the perineum, from which much matter could be pressed. In the centre of the sore, there was a round firm substance, formed by a portion of prolapsed gut; and immediately above this an opening, through which all the feces passed. The parts were seen moving from the pulsations of the femoral artery. Health much impaired, hectic, and the body emaciated to an extreme degree; pulse quick, and so very feeble as not to be reckoned; feet cold. Had been subject to femoral hernia for twelve months: a month before admission, it appears to have been strangulated, and after eight days it sloughed, since which all the feces had passed through the wound.

This case was very unpromising, and, from the excessive debility, it seemed almost hopeless. A nourishing diet, with a liberal allowance of wine and brandy, produced little improvement. The appearance of the discharges soon showed that her debility chiefly arose from inanition, owing to the artificial opening having taken place in a part of the gut where the process of assimilation was still incomplete. With the double purpose, therefore, of affording nourishment, and dilating the lower intestines, I directed the use of large beef-tea enemata three times a day.

The next object was to prevent the escape of the contents of the gut, which not only kept up the debility, but produced sloughing of the sore, and hindered it from healing. The aperture admitted the finger, by which it was ascertained that only one side of the gut had been destroyed, and that the other side formed a continuous surface towards the abdomen. This case, therefore, did not require the application of the ingenious instrument of Baron Dupuytren. Plugging the opening with various conical shaped sponges was first tried; but she could not bear the degree of pressure necessary to retain them *in situ*. Recourse was therefore had to a long cylindrical tent of lint firmly rolled up. This was pushed deeply into the gut, so as completely to fill the external aperture, and in this way nearly to maintain itself in position. Over this graduated compresses were placed, and the whole supported by a light truss. She could not wear any kind of truss, however, more than a few hours at a time, and firm bandaging was substituted for it.

The day after the commencement of this plan, flatus was discharged by the rectum, and this was succeeded by regular and natural evacuations, which continued throughout the treatment.

Her improvement in health was now rapid, and by the end of December the sore had contracted to the size of a shilling, under the application of the chloride of lime lotion, and nitrate of silver in substance.

Still the tent could not be steadily retained without resting its extremity on the posterior surface of the gut, which more or less obstructed the passage, and forced some of the fluid feces outwards by the wound, thus hindering the cicatrization of the sore. An attempt was therefore made to construct an instrument, which might at once fill the external opening, and, by a spring or elastic loop pushed through it, should distend the circle of the gut, and allow the feces to pass onwards to the rectum. This attempt, however, failed, owing to the difficulty of obtaining a substance of proper elasticity; and, on the 16th January, the opening was freely touched with the actual cautery. All discharge ceased for several days, and, after the sloughs separated, considerable contraction took place. The cautery was freely and frequently repeated from time to time during her stay in the house, until the aperture had diminished to a very small point. The discharge was now only occasional, and so trifling as to give little inconvenience. Her health and strength were perfectly restored; and, as she was desirous to go home, I dismissed her, at the end of April, without any further attempt at a complete closure of this minute fistula, which, I believe, becomes the more difficult to be effected the smaller it is.

The actual cautery is very serviceable in many cases. I have cured several fistulæ of the urethra in this way, which had resisted every other means; and I lately saw a patient from whom I removed a very large fungus of the antrum five years ago. Her disease has not returned, solely, I am convinced, owing to the free application of the hot iron. In hemorrhagic oozings from sores or wounds, by which debilitated patients are frequently cut off, the eschar formed in this way is very effectual; and in obstinate bleeding from leech bites, especially in restless children, where pressure is difficult, the point of a red hot wire will at once check the discharge.

The objection to this remedy on account of pain, when compared to other caustics, has been greatly exaggerated, and is by no means verified by the evidence of the patients themselves. The pain is no doubt very acute, but it is transient, and I have never known any of the protracted suffering or subsequent inflammation, which are so frequently seen after the more common caustics. It changes the character of obstinate sores, whether irritable or indolent, and excites in them a new, and generally a healing action. Of this there cannot be a better example than the Onychia Maligna, in almost every stage of which it may be said to be quite a specific.*

* Glasgow Medical Journal.

